# Conference Report: WORKSHOP ON CHARACTERIZING DIAMOND FILMS II Gaithersburg, MD February 24–25, 1993

**DOI:** 10.6028/jres.098.029

**Published:** 1993

**Authors:** Albert Feldman, Charles Beetz, Paul Klocek, Grant Lu

**Affiliations:** National Institute of Standards and Technology, Gaithersburg, MD 20899-0001; Advanced Technology Materials, 7 Commerce Dr., Danbury, CT 06810; Texas Instruments, P.O. Box 655012, Dallas, TX 75265; Norton Diamond Film, Goddard Rd., Northboro, MA 01532

## 1. Introduction

The second in a series of workshops was held at NIST on February 24–25, 1993 to discuss in depth specific topics deemed important to the characterization of diamond films made by chemical vapor deposition (CVD) and to address the need for standards in diamond technology. The topics chosen for this workshop were based on feedback from the attenders of the first workshop [1]. The audience targeted for this workshop was the producers and potential users of CVD diamond technology. University scientists and scientists from government laboratories were invited as experts in properties measurements. There were 44 attenders at the workshop.

We focused on three technical topics for discussion: characterization of optical absorption and scattering for optical applications; electronic characterization–metallization and electronic contacts for electronic applications; and standardization of thermal conductivity measurement. In addition, a short session presented some new developments in Raman measurements and in thermal conductivity measurements.

An important session of the workshop was devoted to the formation of a working group for standardizing thermal conductivity measurements. Grant Lu of Norton Diamond Film was informally chosen to chair the working group. At this session, a round-robin comparison of thermal conductivity and thermal diffusivity measurements was agreed upon. A set of specimens prepared voluntarily by producers of CVD diamond will be circulated among experts in the measurement methodologies. NIST will coordinate the circulation of the specimens among the measurement laboratories and will collate the results of the measurements for presentation at the next working group meeting.

All registrants have been asked to evaluate the workshop. The purpose for this evaluation was to determine the usefulness of the workshop, the desire for further workshops, topics for future workshops, and the need for additional standards related activities. The feedback received will be included in a NIST Internal Report to be distributed to workshop participants.

The sections that follow contain summaries of the main sessions of the workshop. The principal conclusions of the workshop include:

- There is no apparent need for special techniques to optically characterize CVD diamond. Techniques used on other materials appear to be adequate.
- Sessions on optical characterization and electrical characterization should be part of future workshops in order to maintain a free exchange of information among workers in the field.
- A working group to investigate standardization of thermal conductivity measurements on CVD diamond has been formed.
- Planning was begun for an interlaboratoiy round-robin comparison of thermal conductivity and thermal diffusivity measurements. NIST will coordinate this activity.

## 2. Characterization of Optical Absorption and Scattering

Seven presentations that discussed procedures for measuring the optical absorption and scattering in CVD diamond were given.

The first presentation was by P. Klocek of Texas Instruments who discussed and compared the physical properties of diamond with other optical materials. While the broadband transparency, low thermooptic constant, and low dispersion of diamond are very attractive, it is the combination of these properties with exemplary mechanical and thermal properties that make diamond compelling for passive optics, i.e., infrared windows, coatings, geometric optics, diffractive optics. Specific application requirements such as transmission, emission and optical phase were discussed in terms of measurable optical properties, i.e., refractive index *n*, and extinction coefficient *k*, absorption coefficient, scattering coefficient, and modulation transfer function. Microscopic theory (quantum theory of phonons) and macroscopic theory (Maxwell’s equations) were used to introduce the various optical measurement techniques for the optical properties. Tolerances or variances were suggested for these properties for the various passive optical applications along with a simple statistical method for determining the required measurement technique accuracy.

J. M. Trombetta of Texas Instruments presented a discussion of the common methods for determining the infrared absorption coefficient spectrum of an optical material specimen. CVD diamond now under development is thin, typically less than 1 mm thick. This translates into a small material response and therefore low precision in transmission and emission measurements. While background noise is low in Fourier-transform infrared (FTIR) spectrometers, signal drift and reflection artifacts in the instrument cause errors in the absolute transmittance. Photoacoustic spectroscopy can assist in distinguishing absorption loss features from scattering loss but the technique is generally non-quantitative. Therefore, for highly accurate properties determinations, a combination of techniques would be most valuable. Dr. Trombetta indicated that a proper identification of the various contributions to transmission loss requires a complete optical analysis, including measurement of both scattering and absorption spectra.

C. A. Klein of Raytheon, discussed laser damage in diamond. He stated that the laser damage threshold of diamond is high but not uniquely so. While diamond’s exceptionally large figure-of-merit for thermal shock resistance suggests an outstanding laser damage threshold for high average-power optical radiation, its large nonlinear refractive index *n*_2_, suggests a low critical power for self-focusing and, hence, a low peak-power damage threshold. The low self-focusing threshold combined with the occurrence of surface damage due to graphitization will thus limit diamond’s usefulness as a window for transmission of high peak-power laser radiation. Measured data from various sources was reviewed. Type IIa natural diamond crystals exposed to picosecond pulses of 355 nm radiation exhibited surface ablation at threshold intensities of roughly 60 GW/cm^2^ Surface damage (melting) was observed at peak intensities of 6 TW/cm^2^ when diamond was irradiated with femto-second pulses at 620 nm. Single picosecond pulses of 1.06 µm radiation from a Nd:YAG laser resulted in internal damage due to dielectric breakdown. The large value of *n*_2_(2.3 × 10^−13^) resulted in a self-focusing critical power of 1.7 MW and a breakdown intensity of 1 to 3 TW/cm^2^, Another study showed that type IIa diamond, exposed to nanosecond pulses of CO_2_ laser radiation at 10.6 µm, exhibited sub-surface damage at intensities in the low GW/cm^2^ range. The results suggested that the break-down intensity depended on laser wavelength and pulse characteristics. To date, the only laser damage studies reported on CVD diamond were performed on poor quality samples. Laser damage studies are needed on high-quality CVD diamond.

K. A. Snail of the Naval Research Laboratory discussed the need to determine the amount of bulk and surface light scattering in CVD diamond, particularly for infrared window/dome applications. Scattering measurements performed at 0.633 and 10.6 µm on polished specimens of poor quality CVD diamond revealed significant bulk scattering which was much greater than scatter exhibited by typical optical materials. A first order scattering theory was described which allows for distinguishing between bulk scatter and surface scatter. Studies with a microscope equipped FTIR spectrometer suggested that the grain boundaries of the CVD diamond are the major source of bulk scatter. Commercial light scattering equipment capable of measuring the bidirectional distribution function is available; however, its use is limited to only a few laser sources.

R. P. Miller of Raytheon discussed the use of laser calorimetry to measure optical absorption coefficients. The basic techniques for measuring optical absorption were discussed. Transmittance or photometric techniques possess spectral information but lack sensitivity; absorption coefficients not much less than 10^−2^cm^−1^ can be measured with these techniques. Emittance techniques are sensitive to much smaller absorption coefficients (10^−5^cm^−1^) but are subject to background noise. Laser calorimetry is sensitive to small absorption coefficients (10^−5^ cm^−1^) and relatively straight-forward to perform. However, spectral information is limited to the wavelengths of the laser sources used; thus, the technique has limited usefulness if one requires spectral information about materials that exhibit complex absorption spectra. Because CVD diamond is thin, one has difficulty in separating the surface from bulk contributions to absorption. Bulk scattering in the diamond also complicates the calculation of absorption coefficient; if the scattering is not properly taken into account, one can overestimate the absorption coefficient. Data from several sources were discussed. A CO_2_ laser was the optical source. Typical absorption coefficients for natural type IIa diamond were found to be 0.06 cm^−1^ at 9.2 µm and 0.04 cm^−1^ at 10.6 µm, although values as high as 0.4 cm^−1^ at 10.6 µm were found. This may result from sample to sample variability or from slight differences in the wavelength of measurement on a specimen with an absorption coefficient that has a strong wavelength dependence. (CO_2_ lasers can operate at several closely spaced wavelengths such as 10.591 and 10.67 µm.) Data at local spots in high quality CVD diamond showed absorption coefficients of 0.094 cm^−1^ at 9.27 µm and 0.067 cm^−1^ at 10.591 µm, without correction for surface absorption which was believed to be small.

L. H. Robbins of NIST discussed the use of specular transmittance and reflectance to measure the optical constants of CVD diamond. Expressions for transmittance and reflectance in the presence of surface scatter and bulk absorption were presented. By fitting the measured transmittance and reflectance to these expressions, values are obtained for the sample thickness, root-mean-squared surface roughness, refractive index and extinction or absorption coefficient. The model was developed for the study of thin unpolished optical films, less than several micrometers thick, where the surface scatter only partially attenuates the specular beams. This corresponds to a surface roughness to wavelength ratio close to unity. Transmittance and reflectance measurement on CVD diamond samples 0.42 µm thick with surface roughness 10 nm, and 1.90 µm thick with surface roughness 30 nm were fit to the model. The calculated absorption coefficients were significantly higher than the absorption coefficients of type IIa diamond. Because most CVD diamond material produced is much thicker than the specimens measured, the typical roughnesses will be much greater. For these types of specimens, unless they are polished, the model has limited usefulness in the ultraviolet-visible range, but the model may prove useful in the infrared.

In the final presentation, M. E. Thomas of Johns Hopkins University discussed how emissivity measurements can be used to obtain optical properties of diamond. In the two-phonon region, 4 to 7 µm, diamond has a fairly high absorptivity and therefore a high emissivity. The emissivity of diamond in this region was found to be 0.8 and not very sensitive to temperature; thus, the emission signal is large. In the three- and four-phonon regions, 2 to 4 µm, diamond is weakly emissive. However, if the specimen is sufficiently thick, the emitted radiation in this spectral band can have a useful signalmoise ratio. Thomas pointed out that in the 7 to 12 µm region, which is of great interest, emissivity measurements are difficult to make because the background emission in this spectral band is large relative to the diamond emission. Because the optical phonon spectrum in diamond begins at wavelengths below this spectral range, emission is exceedingly weak and the signalmoise ratio is small. Work is now in progress to overcome this difficulty. Other difficulties with emission measurements on diamond include oxidation above 800 K when high temperature measurements are performed and light scattering within the sample. At this time, emission measurements on CVD diamond appear to be most useful in the 2 to 7 µm region and perhaps in the visible region in low scatter specimens. However, measurement of absorption by emissivity measurements in the 7 to 12 µm region is difficult.

In summary, the session on optical characterization of CVD diamond benefitted both speakers and other participants because of the free exchange of information.

## 3. Electronic Characterization—Metallization and Electronic Contacts

Seven speakers presented different aspects of the electrical properties of semiconducting diamond and the formation of ohmic and Schottky contacts.

The first paper was given by J. Vandersande from the Jet Propulsion Laboratory. He presented the results of recent electrical resistivity measurements on diamond between room temperature and 1200 °C. He described a special apparatus designed for making resistivity measurements up to 1200 °C and showed that resistivity values of ~10^16^Ω cm typically reported in the literature for type IIa natural diamond represent a lower limit to the true values because of limitations in the measurement instrumentation. The resistivity of type IIa diamonds was found to drop from ~10^15^Ω cm at 200 °C to 10^4^Ω cm at 1200 °C, with an activation energy of ~1.5 to 1.6 eV. Results were also presented for CVD diamond films that showed electrical resistivities comparable to or greater than those for natural type IIa diamonds; the CVD films also had similar activation energies. Dr. Vandersande cautioned that during high temperature measurements, surface graphitization can occur, leading to a decrease in specimen resistance on cooling. The surface graphitization layer can be removed by cleaning in acid solutions, thus restoring the original specimen resistance.

C. Hewett from the Naval Command, Control and Ocean Surveillance Center discussed the importance of low resistance ohmic contacts to high power device applications of semiconducting diamond. Ohmic contact formation is based on a solid state reaction in which a transition metal reacts with the diamond to form a carbide layer during high temperature annealing. This carbide interface layer is in intimate contact with both the metal contact and the underlying diamond, thus promoting good adhesion between the metal contact to the diamond. Dr. Hewett described a technique for measuring the specific contact resistance using a circular transmission line model. This technique eliminates the need for mesa etching, a procedure that is usually needed for minimizing artifacts due to three dimensional current flow.

M. Geis of Lincoln Laboratories reviewed the device properties of diamond and discussed the advantages and disadvantages of diamond in high power, high frequency devices. He discussed the use of various surface treatments for passivating the diamond surface in order to eliminate surface leakage currents. Exposing diamonds to CF_4_O_2_ or N_2_ plasmas gave the best results. Dr. Geis also presented interesting results which illustrated that artifacts in capacitance-voltage measurements can be caused by back surface contact resistance. He also discussed interesting approaches to achieving large area single crystal diamond substrates; a novel method was developed for placing of highly oriented diamond seed crystals on patterned Si substrates. He also discussed applications of diamond as a cold electron emitter.

J. von Windheim from Kobe Steel USA, Inc., discussed electronic transport measurements on natural single crystal diamonds, homoepitaxial CVD diamond, and polycrystalline CVD diamond films. He reviewed the results of various measurements such as high temperature resistivity, ac conductivity, dc current-voltage measurements, and space charge limited currents. In addition, he described the role of these measurements in identifying various trapping centers. Results of Hall effect and resistivity measurements indicate a complex conduction process in which 2 to 20 percent of the boron atoms used to dope the diamond specimens are compensated. The nature of the compensating center has not been elucidated but it is believed to be nitrogen, possibly in the A-aggregate form. Comparisons were made between the transport properties of polycrystalline diamond films and diamond single crystals. The transport properties of the polycrystalline films were much poorer than those of the single crystals.

J. Glesener of the Naval Research Laboratory presented additional results describing the electrical characterization of impurities in diamond. He employed admittance spectroscopy to characterize deep levels associated with boron impurities. The technique consisted of measuring the ac conductivity of a Schottky-barrier diode fabricated on the diamond. The conductance of the sample was measured as a function of temperature and as a function of the ac frequency. The ac voltage applied to the Schottky diode modulates the inter-section of the Fermi level with the impurity level, providing a time dependent source of carriers. Dr. Glesener showed that the conductance peaked when the carrier emission rate from the trap became comparable to the ac frequency. The trap emission rate could be changed by lowering the temperature of the diamond. From these measurements, the energy level of the deep trap was found to be 0.33 eV and the hole capture cross-section was estimated to be ~2×10^−12^ cm^2^ The deep trap was believed to be due to boron that had been present in the gas used to grow the diamond.

K. Das from Kobe Steel USA, Inc. discussed contacts on diamond. He reviewed the results for a wide range of metals, semiconductors, silicides and metal carbides as rectifying contacts on semiconducting diamond. He showed that direct metallization on CVD grown films does not always produce good rectifying contacts; it is sometimes necessary to introduce an insulating diamond or dielectric film between the doped diamond and the metal in order to get a good rectifying characteristic. He also showed that the rectification properties of metal contacts can be improved by near surface implantation of boron into the diamond prior to the metallization step. Dr. Das also discussed the formation of low resistance ohmic contacts using carbide forming transition metals deposited on heavily doped diamond films. He also presented data on the chemical composition of the formed contact structures.

The last presentation was given by C. P. Beetz of Advanced Technology Materials, Inc. He discussed the measurement of Schottky-barrier heights of metals on diamond using the method of internal photoemission. He listed the Schottky barrier heights of various metals on diamond and discussed the observation of a region showing two thresholds in the photo-yield of metals on type IIa natural diamond. Dr. Beetz pointed out that the upper threshold values presented in some of the literature must be corrected to take into account contributions from the lower threshold. He also discussed controlling the Schottky-barrier height of metals on diamond by using shallow near surface silicon implants. These implants would favor reactions with silicide forming metals. He showed results for platinum, molybdenum, and titanium contacts in which the barrier height decreased with increasing silicon implant dose.

## 4. Work in Progress and New Developments

A special session was organized for presentation of new resuhs and to cover miscellaneous topics not covered by the principal sessions.

E. Etz of NIST presented recent results of Raman spectroscopy measurements on several specimens provided by two producers of CVD diamond. This work was presented in the context of a proposed Raman standard reference material discussed at the previous workshop. The Raman spectrum is being used as a measure of diamond quality. The quality of diamond is considered to decrease with increasing line width of the diamond Raman line at 1332 cm^−3^, increasing background luminescence intensity, and increasing intensity of the *sp*^2^ carbon peak near 1550 cm^−1^. In high quality films, the *sp*^2^ peak may not be observable. Freestanding diamond wafers of high quality with thicknesses ranging from 300 μm to 1.7 mm were examined. A cross-sectional examination by Raman spectroscopy indicated that the diamond in the bottom layer (the material closest to the substrate during deposition) was of poorer quality than the diamond in the top layer. The width of the diamond Raman line in one of the specimens was equal to the width of the diamond Raman line in a type IIa single crystal natural diamond. This result suggests that the specimen was essentially strain free. The Raman spectrum of a specimen deliberately doped with nitrogen indicated a deterioration in the quality of the diamond. A trace analysis of a cross-section indicated the presence of Na, K, Ca, and Mg, possibly due to contamination of the specimen during preparation.

At the previous workshop, D. Morelli of the General Motors Research Laboratory discussed the thermal conductivity of CVD diamond as a function of temperature. The curve representing the temperature dependence at low temperature showed a kink that could not be explained satisfactorily. At this workshop. Dr. Morelli presented the results of an experiment that appears to explain the effect. In this experiment, thermal conductivity measurements were made on single crystal diamond specimens before and after exposure to neutron radiation. Prior to irradiation, the specimens did not exhibit the kink in the thermal conductivity whereas after irradiation and heat treatment, a kink appeared. The temperature at which the kink occurred was correlated with the expected sizes of the defects produced. At low temperatures, the mean wavelength of thermal phonons is greater than the size of the defect so that scattering is reduced. As the temperature increases, the mean phonon wavelength decreases and scattering increases resulting in a decrease in the expected thermal conductivity behavior. The result is a kink in the dependence of thermal conductivity on temperature. The larger the size of the defect, the lower the temperature at which the kink will occur. The work also demonstrated that grain boundaries are transparent to phonon propagation, at least at the low temperatures.

L. Wei of Wayne State University described the method of photothermal deflection for measuring the thermal diffusivities of diamond. Measurements had been performed on a large number of diamond specimens over the last several years at Wayne State. Dr. Wei discussed the measurement of thermal diffusivity as a function of temperature of diamond crystals containing higher ratios of C^12^:C^13^ than the natural abundance ratio. She found that the thermal conductivity increased with decreasing C^13^ content. The temperature dependence of the thermal conductivity could be explained by a theory that took into account both Umklapp phonon scattering processes and normal phonon scattering processes.

## 5. Thermal Conductivity of Diamond: Standardization Issues

Six presentations were given that discussed general standardization issues and specific measurement techniques related to measuring thermal conductivity. R. Tye from Ulvac Sinku-Riko reviewed the ideal requirements for a standard measurement method, standard methods now in use for determining thermal conductivity, and related international standards. Some of the ideal requirements for a standard method are: a generally accepted and proven technique; the availability of a related current standard; relative simplicity in the concept, design and operation of the equipment; rapid and minimal specimen preparation; a capability of providing absolute values to a known precision or a required precision. The advantages and disadvantages of techniques such as axial rod heat flow, 3 omega, flash, converging wave, and ac calorimetry were discussed. The flash method is used in three international standards (ASTM, UK/BS and Japan/JIS) for thermal diffusivity measurements. However, these standards would need modification to be applicable to CVD diamond.

R. Taylor from the Thermophysical Properties Research Laboratory at Purdue University stressed the difficulty of accurately measuring thermal transport properties. He showed that published thermal conductivity values of 99.9 + % pure tungsten and TiC show very large variations; values differing by factors of two to three were observed. Professor Taylor then described a new measurement technique developed at Purdue University. In this technique, a thin strip of diamond 50 mm long×4mm wide is partially masked. The unmasked portion is uniformly exposed to a step input of heat from an infrared lamp by means of a shutter. Three thermocouples, attached along the length of the masked portion of the sample, monitor the rise in temperature as heat flows from the unmasked portion of the specimen to the heat sink at the other end. The time dependence of the temperature is used to compute the thermal diffusivity.

D. Slutz from General Electric Superabrasives discussed the development of two methods suitable for quality control monitoring of thermal conductivity rather than for obtaining absolute accuracy. The first is based on Ångström’s method [2]. A modulated laser beam focused to a line at one edge of the specimen provides the heat source. An infrared detector monitors the phase lag of the traveling thermal wave as a function of the distance from the heating source. The thermal diffusivity is calculated from the data. In the second method, the specimen is heated in the same manner as above but data is collected at a single point on the specimen. A Fast Fourier Transform analyzer is used to analyze the collected data, from which the thermal diffusivity is calculated. The measurement time for each specimen is 20 min.

J. Graebner of AT&T Bell Laboratories described measurement techniques to measure the thermal conductivity in the plane of a specimen and the thermal diffusivity perpendicular to the plane of the specimen. The in-plane measurement is performed with a two-heater steady state technique; one of the heaters allows for a correction due to radiative heat loss and conductive heat loss through the electrical leads. The perpendicular thermal diffusivity is measured by the laser flash technique. A thermal conductivity of 26 W/(cm K) has been calculated from laser flash measurements on high quality CVD diamond.

S. Preston described laser flash measurements of thermal diffusivity at AEA Technology in Great Britain. A high power Q-switched ruby laser was used as the heating source. A relatively thick metallic coating (10 μm thick) was deposited on the diamond to absorb the laser beam without being ablated away. In order to account for the transient delay due the coating, a measurement was performed on a piece of high purity copper foil coated along with the diamond films. Because the thermal diffusivity of the copper foil was known, he was able to calculate the thermal diffusivity of the coating; this value was used in calculating the thermal diffusivity of the diamond.

G. Lu of Norton Diamond Film described the results of thermal modelling to determine the relative importance of in-plane thermal conductivity vs perpendicular thermal conductivity for lowering the temperatures of junctions of diamond with other materials. In most cases, a higher perpendicular thermal conductivity led to a lower junction temperature. The one exception was the case of a small circular heat source having a diameter much less than the diamond thickness; in this case, in-plane heat spreading plays the major role in removing heat from the junction region. Results were also presented which showed that incremental decreases in junction temperature were minimal when the diamond thermal conductivity increased beyond three to four times the thermal conductivity of the heat sink on which it is placed.

## 6. Organization of Working Group

Thermal management applications are leading the way in large-scale commercialization of CVD diamond. Recent press announcements [3] show the incorporation of CVD diamond into the production of high power electronic packages. However, not only is there no accepted method of measuring the thermal conductivity of CVD diamond, but different methods can frequently yield significantly different results. Therefore, a working group has been formed to examine the issues and formulate a recommendation on standardizing thermal conductivity measurements of CVD diamond. The members of the working group are:

Grant Lu, Norton Diamond Film–Chairman
Albert Feldman, NIST
John Graebner, AT&T
Ronald Tye, ULVAC Sinku-Riko
David Slutz, General Electric
Pao-Kuang Kuo, Wayne State University
Steven Preston, AEA Technology–Associate Member

As one of the first steps in the process, an interlaboratory round-robin test will be conducted to assess differences in the results of measurements made at different laboratories using different methods. A set of samples will be fabricated by several manufacturers and sent to different laboratories for measurement of thermal conductivity. At the workshop, Norton Diamond Film, General Electric, and Raytheon agreed to supply specimens. AT&T, Wayne State University, Purdue University, AEA Technology, Sinku Riko, General Electric, and Norton Diamond Film offered to perform the measurements. Other manufacturers and measurement laboratories will be asked to participate. The results will be compiled by NIST and presented at the next workshop.

## References

[1] Feldman A, Beetz CP, Droiy M, Holly S (1992). Workshop on Characterizing Diamond Films.

[2] Ångström MA (1863). A New Method of determining the Thermal Conductibility of Bodies. Phil Mag.

[3] (1992). Photonics Spectra.

